# Utility of testing for apraxia and associated features in dementia

**DOI:** 10.1136/jnnp-2015-312945

**Published:** 2016-06-01

**Authors:** Samrah Ahmed, Ian Baker, Sian Thompson, Masud Husain, Christopher R Butler

**Affiliations:** 1 Nuffield Department of Clinical Neurosciences, University of Oxford, John Radcliffe Hospital, Oxford, UK; 2 Russell Cairns Unit, Oxford University Hospitals NHS Trust, John Radcliffe Hospital, Oxford, UK; 3 Department of Clinical Neurology, Oxford University Hospitals NHS Trust, Oxford, UK; 4 Department of Experimental Psychology, University of Oxford, John Radcliffe Hospital, Oxford, UK

## Abstract

**Introduction:**

Existing literature suggests that the presence or absence of apraxia and associated parietal deficits may be clinically relevant in differential diagnosis of dementia syndromes.

**Aim:**

This study investigated the profile of these features in Alzheimer's disease (AD) and frontotemporal dementia (FTD) spectrum disorders, at first presentation.

**Methods:**

Retrospective case note analysis was undertaken in 111 patients who presented to the Oxford Cognitive Disorders Clinic, Oxford, UK, including 29 amnestic AD, 12 posterior cortical atrophy (PCA), 12 logopenic primary progressive aphasia (lvPPA), 20 behavioural variant FTD (bvFTD), 7 non-fluent variant PPA (nfvPPA), 6 semantic variant PPA (svPPA) and 25 patients with subjective cognitive impairment (SCI). The clinical features of interest were: limb apraxia, apraxia of speech (AOS), and left parietal symptoms of dyslexia, dysgraphia, and dyscalculia.

**Results:**

The prevalence of limb apraxia was highest in PCA, amnestic AD, lvPPA and nfvPPA. AOS was only observed in nfvPPA. Associated parietal features were more prevalent in AD spectrum than FTD spectrum disorders. Group comparisons between key differential diagnostic challenges showed that lvPPA and nfvPPA could be significantly differentiated on the presence of left parietal features and AOS, and amnestic AD could be differentiated from bvFTD, svPPA and SCI by limb apraxia. Regression analysis showed that limb apraxia could successfully differentiate between AD and FTLD spectrum disorders with 83% accuracy.

**Discussion:**

Disease-specific profiles of limb apraxia and associated deficits can be observed. FTD and AD spectrum disorders can be difficult to differentiate due to overlapping cognitive symptoms, and measures of apraxia, in particular, appear to be a promising discriminator.

## Introduction

Apraxia is a disorder of higher-order motor skills and learned movements, in the absence of paresis, abnormal muscle tone, cerebellar ataxia sensory impairment or comprehension deficits.^1^ ^2^ Apraxia has been associated with various dementia syndromes but, although simple to test for at the bedside, its use in the differential diagnosis of dementia is not well established. Several forms of apraxia are often distinguished, such as constructive apraxia, gait apraxia and trunk apraxia. The clinical manifestations relevant to this study are those affecting limb movements. Limb apraxia is typically described in terms of two major types: ideomotor apraxia refers to difficulty pantomiming learned actions such as brushing the teeth or combing the hair; ideational apraxia describes an inability to carry out complex sequences of actions in everyday life, such as making a cup of tea.^3^

A survey of the existing literature reveals common observations of apraxia in amnestic Alzheimer's disease (AD). Apraxia is a diagnostic feature of amnestic AD, included in the NINCDS-ADRDA (National Institute of Neurological and Communicative Diseases and Stroke-Alzheimer's Disease and Related Disorders Association) criteria.^4^ ^5^ Ideomotor and ideational apraxia have been reported in amnestic AD,^6^ with the former being reported as being more prevalent, although not ubiquitous.^7^ Limb apraxias have also been observed in the clinical variants of AD, including posterior cortical atrophy (PCA)^8^ and logopenic variant primary progressive aphasia (lvPPA).^9^ ^10^

The prevalence of apraxia has been less systematically examined in frontotemporal dementia (FTD) disorders of semantic (svPPA) and non-fluent variant PPA (nfvPPA), or behavioural variant FTD (bvFTD). Limb apraxia has been observed in a substantial minority of patients with nfvPPA,^11^ ^12^ although the defining feature of apraxia of speech (AOS), an acquired speech disorder which affects the motor programming system required for speech production,^13^ was more consistently found.^12^ In this group, limb apraxia occurred with greater frequency than in patients with lvPPA.^11^ A more recent study found that patients with amnestic AD performed significantly worse than patients with bvFTD on tests of limb apraxia, examining both ideational and ideomotor gestures.^14^

It is widely held that the left parietal lobe plays a central role in limb apraxia, originating in Leipmann's early model describing a posterior to anterior stream that converts mental images of actions into a motor response,^2^ and subsequent models have equally attributed a central role to the left parietal regions.^1^ ^15^ In conjunction with apraxia, left parietal dysfunction produces a range of other cognitive symptoms, including deficits in reading, writing and arithmetic, and there is suggestion in the literature that these clinical features may also be of relevance to the diagnosis of dementia syndromes, particularly in the AD spectrum. The neuropsychological profile of patients with lvPPA, where pathology is focused on the left inferior parietal lobe, includes dyscalculia, and phonological alexia as well as limb apraxia^10^ and dysgraphia.^16^ ^17^ In a series of 19 patients with PCA, McMonagle *et al*^8^ reported that 18 cases had dyslexia or dysgraphia while 16 had dyscalculia.

Although the existing literature suggests that the presence or absence of apraxia and associated parietal deficits might be clinically relevant in differential diagnosis, to date there has, to our knowledge, been no systematic examination of apraxia and associated features in a large number of patients with different dementia syndromes. The aim of this study is to determine the profile of these features in AD and FTD spectrum disorders, at initial clinical presentation.

## Methods and materials

### Participants

All patients were assessed at clinical presentation at the Oxford Cognitive Disorders Clinic, Oxford, UK. A total of 111 patients were entered into the study with the following clinical diagnoses: 29 amnestic AD, 12 PCA, 12 lvPPA, 20 bvFTD, 7 nfvPPA and 6 svPPA. All patients recruited for the study fulfilled consensus criteria for disease (PCA;^8^ ^18^ lvPPA, nfvPPA and svPPA;^13^ amnestic AD;^4^ ^5^ or bvFTD^19^), based upon clinical assessment, brain imaging and detailed neuropsychological assessment. Case notes for all patients seen in the Oxford Cognitive Disorders Clinic between 2010 and 2015 who had a diagnosis of an AD (amnestic AD, lvPPA, PCA) or FTD (bvFTD, svPPA or nfvPPA) spectrum disorder were examined. Mean follow-up time for patients was 22.7 months; 25 patients with subjective cognitive impairment (SCI) were also included as a control group, as well as an important clinical comparison group. Patients with SCI presented with SCI, no objective cognitive impairment on neuropsychological screening tests (within 1.5 SDs of normal subjects), no evidence of focal atrophy on an MRI or CT scan (where available), and no neurological explanation for their cognitive symptoms being identified.

All patients were assessed by a senior behavioural neurologist (CRB, ST or MH), and underwent a clinical interview, neurological examination, neuropsychological screening and further investigations where necessary, to reach a clinical diagnosis. Patients were administered a baseline cognitive screening assessment, the Addenbrooke's Cognitive Examination-Revised (ACE-R). The ACE-R consists of subsections testing memory, attention, fluency, language and visuospatial skills. Patients underwent MRI (or CT scan where MR was contraindicated, e.g., by a pacemaker).

### Retrospective case note analysis

At the first assessment in the Cognitive Disorders Clinic, presenting symptoms described in the clinical interview with the patient and carer, and signs identified during a standard neurological examination^3^ were systematically recorded in the case notes by the examining clinician. For this study, clinical notes were examined by a neuropsychologist (SA) not involved in the initial evaluation and diagnosis of patients. Clinical features were coded as being ‘present’ or ‘absent’ at initial presentation in three main categories of interest:

1. Limb apraxia: As in Kas *et al*,^20^ limb apraxia scores pooled reference to the imitation of meaningless, meaningful and pantomime of familiar gestures, on the basis that these actions are of localisation value, consistent with a central role for the left parietal lobe in the integration of limb praxis information.^15^ ^20^ ^21^ Patients were asked to: (A) pantomime the use of a tool on verbal command, for example, ‘show me how you would brush your hair’ or ‘pretend to strike a match and blow it out’; (B) imitate the clinician creating meaningless gestures and postures using the left and right hands; (C) pantomime the sequential steps involved in performing a complex task on verbal command, for example ‘show me how you would make a cup of tea’.
2. Speech articulation: Apraxia of speech was defined according to published criteria,^13^ which include effortful, halting speech with the presence of speech sound errors and distortions.
3. Associated left parietal features: Existing literature points to three features of interest: dysgraphia, defined as impairment of well-formed and linguistically correct script; dyslexia, defined as a disturbance in the ability to read and spell; and dyscalculia, defined as an impairment in the ability to comprehend or write numbers properly, or to manipulate numbers to perform simple calculations.^3^ Corroboration of the presence or absence of these features was determined from the ACE-R, where available.

### Statistical analysis

Group differences in demographic and clinical characteristics were examined using one-way analysis of variance. Group comparisons of clinical features were examined using Fisher's exact test. Binary logistic regression was used to determine if any of the clinical features could be used to predict diagnostic category. Cross validation of the regression analyses was undertaken by taking a random sample of half of the patients from the original sample, and repeating the binary logistic regression analyses.

## Results

### Demographic and clinical characteristics

The demographic and clinical characteristics of patients are shown in table 1. There were no significant between group differences in age, with the exception of those with lvPPA, amnestic AD and svPPA, compared to patients with SCI, where such patients were significantly younger. There were no significant between group differences in education levels. Since the disorders studied present with a range of cognitive deficits that are given differential weighting in the baseline cognitive screening task used (the ACE-R), patients were matched on symptom duration, that is, time since the first symptom was noticed.

**Table 1:** Demographic and clinical characteristics of patients at initial presentation

Clinical characteristics	amnestic AD (n=29)	PCA (n=12)	lvPPA (n=12)	bvFTD (n=20)	nfvPPA (n=7)	svPPA (n=6)	SCI (n=25)
Age (years)	63.9^a^ (8.4)	61.4 (6.2)	69.2^c^ (8.7)	61.7 (8.4)	67.0 (7.6)	68.2^a^ (7.6)	57.4 (7.2)
Education (years)	13.3* (2.7)	11.9 (1.9)	12.1 (2.5)	12.8 (2.6)	12.7 (2.6)	12.7 (2.7)	13.2 (2.8)
Symptom duration (years)	2.3* (1.3)	2.2 (0.79)	2.2* (0.85)	2.6 (2.2)	2.6 (2.1)	1.6 (0.77)	2.8 (2.8)
ACE attention and orientation (18)	12.9^d^ (3.4)	13.8^a^ (3.9)	12.4 (5.9)	14.8 (3.6)	16.6 (3.1)	17.8 (0.5)	17.7 (0.47)
ACE memory (26)	11.6^d^ (4.2)	16.3^b^ (6.8)	10.9^d^ (9.2)	15.1^d^ (6.0)	19.0 (6.6)	17.8 (4.2)	23.5 (3.2)
ACE fluency (14)	6.3^d^ (3.1)	8.6 (3.8)	6.0^c^ (4.8)	5.1^d^ (3.6)	5.2^b^ (3.8)	6.5 (1.3)	11.6 (1.9)
ACE language (26)	22.0^a,h^ (3.8)	20.3^b^ (4.1)	15.7^d^ (6.8)	20.2^c^ (4.9)	20.8 (4.0)	16.5^b^ (2.9)	25.7 (0.66)
ACE visuospatial (16)	11.2^d,e^ (3.9)	7.6^d^ (4.8)	12.9^f^ (3.0)	13.4^g^ (2.9)	15.8^g^ (0.5)	15.8^g^ (0.5)	15.9 (0.49)
ACE total (100)	65.5^d^* (14.5)	66.7^d^ (16.5)	57.6^d^* (27.0)	69.0^d^* (16.2)	76.8* (15.5)	74.5* (6.0)	94.6 (3.3)

*Significant difference* compared to ^a^SCI p<0.05; ^b^SCI p<0.01; ^c^SCI p<0.001; ^d^SCI p<0.0001; ^e^PCA p<0.05; ^f^PCA p<0.01; ^g^PCA p<0.0001; ^h^lvPPA p<0.001.

*Missing data: Education level not recorded for 3 patients with amnestic AD; symptom duration not available for 1 lvPPA and 1 patients with amnestic AD as they presented alone; ACE not completed in 2 lvPPA, 1 amnestic AD, 1 bvFTD, 2 nfvPPA and 2 svPPA due to either refusal by patient or clinicians decision not to test due to patient severity/anxiety.

ACE, Addenbrooke's Cognitive Examination; AD, Alzheimer's disease; bvFTD, behavioural variant frontotemporal dementia; lvPPA, logopenic variant primary progressive aphasia; nfvPPA, non-fluent variant primary progressive aphasia; PCA, posterior cortical atrophy; SCI, subjective cognitive impairment; svPPA, semantic variant primary progressive aphasia.

On the cognitive subscores obtained from the ACE-R screen, there were significant differences between SCI and the patient groups in all cognitive domains including the total ACE-R score. Patients with PCA were significantly more impaired on visuospatial skills compared to all other groups, and patients with lvPPA had significantly poorer language compared to those with amnestic AD.

### Apraxia and associated features

The prevalence of limb apraxia was highest in PCA (91.7%), followed by almost equal prevalence in amnestic AD and lvPPA (69% and 66.7%, respectively). More than half the patients with nfvPPA (57.1%) also had limb apraxia (table 2). AOS was only observed in nfvPPA, present in all but two patients. Overall, associated parietal features were more prevalent in the AD spectrum than FTD spectrum disorders, being highest in lvPPA followed by patients with PCA and amnestic AD. More specifically, dysgraphia and dyslexia were more prevalent in lvPPA, while dyscalculia was more prevalent in PCA. Associated parietal features were entirely absent in svPPA. Limb apraxia was absent in SCI, and evidence of dysgraphia and dyslexia were found in one patient only.

**Table 2:** Percentage of patients with each feature at initial presentation

	AD spectrum			FTD spectrum			Control group
Observed at initial presentation (%)	amnestic AD (n=29)	PCA (n=12)	lvPPA (n=12)	bvFTD (n=20)	nfvPPA (n=7)	svPPA (n=6)	SCI (n=25)
Apraxia							
Limb	69.0	91.7	66.7	10	57.1	0	0
AOS	0	0	0	0	71.4	0	0
Associated left parietal features*	34.5	50	75	15	14.3	0	4
Dysgraphia	20.7	33.3	75	15	14.3	0	4
Dyslexia	6.9	8.3	33.3	0	0	0	4
Dyscalculia	13.8	41.7	16.7	0	0	0	0

*Percentage of patients with at least one feature.

AD, Alzheimer's disease; AOS, apraxia of speech; bvFTD, behavioural variant frontotemporal dementia; FTD, frontotemporal dementia; lvPPA, logopenic variant primary progressive aphasia; nfvPPA, non-fluent variant primary progressive aphasia; PCA, posterior cortical atrophy; SCI, subjective cognitive impairment; svPPA, semantic variant primary progressive aphasia.

Group comparisons were run between key differential diagnostic challenges (table 3). Patients with lvPPA and nfvPPA could be significantly differentiated on the presence of associated left parietal features (p<0.05), in particular, dysgraphia (p<0.05) and the presence of AOS (p<0.01). There was a significant difference in the prevalence of limb apraxia between patients with amnestic AD and bvFTD (p<0.0001), svPPA (p<0.01) and SCI (p<0.0001). Patients with amnestic AD also showed significantly more left parietal features than those with SCI (p<0.01).

**Table 3:** Group comparisons between key differential diagnostic challenges

Group comparison	Significantly different presenting symptoms	p Value
lvPPA × nfvPPA	lvPPA >nfvPPA:	
	Dysgraphia	<0.05
	Overall presence of left parietal features	<0.05
	nfvPPA >lvPPA:	<0.01
	AOS	<0.01
amnestic AD × bvFTD	amnestic AD >bvFTD:	
	Limb apraxia	<0.0001
amnestic AD × svPPA	amnestic AD >svPPA:	
	Limb apraxia	<0.01
amnestic AD × SCI	amnestic AD >SCI:	
	Limb apraxia	<0.0001
	Overall presence of left parietal features	<0.01

AD, Alzheimer's disease; AOS, apraxia of speech; bvFTD, behavioural variant frontotemporal dementia; lvPPA, logopenic variant primary progressive aphasia; nfvPPA, non-fluent variant primary progressive aphasia; SCI, subjective cognitive impairment; svPPA, semantic variant primary progressive aphasia.

Since apraxia is a core feature of corticobasal syndrome (CBS),^22^ which is a pathologically heterogeneous syndrome not fitting clearly into AD or FTD spectrum disorders,^23^ we reviewed the records to identify whether any patients subsequently developed additional Parkinsonian features suggestive of CBS. Parkinsonism became apparent in 5/111 patients during follow-up. These patients were diagnosed with bvFTD (n=3), nfvPPA (n=1) and amnestic AD (n=1) at clinical presentation. None of these 3 bvFTD patients nor the nfvPPA patient showed limb apraxia at presentation or follow-up. The amnestic AD patient, who demonstrated apraxia on initial presentation, began to show signs of Parkinsonism only after 24 months of follow-up.

### Prediction of diagnostic category

A binary logistic regression was conducted to predict diagnostic category, using limb apraxia and associated features as predictors. Diagnostic groups were collapsed into AD spectrum (patients with amnestic AD, lvPPA and PCA) and FTD spectrum (patients with bvFTD, svPPA and nfvPPA) disorders. Regression analysis showed that only the presence of limb apraxia was able to reliably discriminate between the groups (OR 0.56, 95% CI 0.014 to 0.226, p<0.0001). Prediction success overall was 82.6%, 83% for AD spectrum disorders and 81.8% for FTD spectrum disorders. Cross validation of the binary logistic regression in half of the patient sample (n=43) confirmed limb apraxia to be a reliable discriminator (odds ratio 0.53, 95% CI 0.106 – 0.507, p<.01). Overall prediction accuracy was also similar (81.4%), although FTD spectrum disorders (93.3%) were more reliably identified than AD spectrum disorders (75%).

## Discussion

The findings of this study show, for the first time, that simple clinical examination for the presence of apraxia and associated left parietal features at initial presentation can assist in differential diagnosis of AD and FTD spectrum disorders that typically show overlapping features.

Limb apraxia and associated left parietal features were most common in the AD spectrum disorders. All but one patient with PCA presented with limb apraxia, and more than two-thirds of patients with amnestic AD and lvPPA were also impaired. Prevalence of associated left parietal features was highest in lvPPA, present in 75% of the group, followed by 50% in patients with PCA, and a third of patients with amnestic AD. These clinical features have been observed in previous studies,^8^ ^12^ ^16^ ^17^ but have not been systematically compared across clinical groups.

Despite the prevalence of limb apraxia and selected parietal features in AD spectrum disorders, the relevance of these symptoms to diagnosis has received very little attention in the literature. The fact that limb apraxia, in particular, was able to differentiate AD spectrum disorders from FTD spectrum with 83% accuracy, is potentially valuable. Several models of apraxia have been proposed, but they all agree that the parietal lobe occupies a central position in the stream of processing implemented in the production of meaningful and meaningless gestures.^15^ Our findings are, therefore, particularly relevant to recent evidence of anatomical overlap between lvPPA, PCA and amnestic AD, showing that all three patient groups are associated with atrophy in the left temporoparietal region, with additional, syndrome-specific, grey matter atrophy in smaller areas.^22^ The examination of imaging data for each patient was outside the scope of this study, but studies have shown that limb apraxia in PCA^20^ and amnestic AD^23^ correlates with hypoperfusion in structures in the left posterior parietal cortex. The identification of common areas of cognitive impairment may, therefore, be particularly useful as an in vivo clinical marker of AD pathology regardless of the main presenting clinical symptoms.

In the FTD spectrum, by contrast, apraxia and associated features were entirely absent in svPPA and were few in bvFTD. Differentiation of svPPA and bvFTD from amnestic AD is a common diagnostic challenge due to overlapping impairment in episodic memory in bvFTD^24^ and word-finding difficulties in svPPA.^25^ Johnen *et al*^14^ ^26^ also recently showed that amnestic AD patients had poorer praxis scores compared to bvFTD on detailed neuropsychological tests of limb apraxia.

Interestingly, limb apraxia was observed in 57% of patients with nfvPPA, a prevalence similar to that in patients with lvPPA and amnestic AD. Without pathological confirmation, the possibility remains that patients with AD pathology were included in this group. However, limb apraxia has been previously documented in nfvPPA. Adeli *et al*^11^ reported significantly greater ideomotor apraxia in patients with nfvPPA compared to those with lvPPA, associated with grey matter loss in the left lateral premotor cortex. No studies have examined the anatomical correlates of limb apraxia in lvPPA to date, though it is likely that the typical pathology in the left parietal cortex will be implicated, consistent with existing anatomical association between limb apraxia and the left parietal lobe.^15^ While there may be differences in the anatomical correlates of apraxia in lvPPA and nfvPPA, clearly our findings suggest caution is needed before ubiquitously equating limb apraxias with AD pathology. Instead, our results point to the clear relevance of taking into account key diagnostic deficits in speech articulation in nfvPPA,^13^ and also in the presence of associated left parietal deficits in reading, writing and arithmetic. These features were almost absent in patients with nfvPPA at clinical presentation, compared with frequent presence in patients with lvPPA.

There are limitations to this study. The brevity of case notes and the retrospective nature of case note analysis prevents further comment on severity or duration of features. In addition, while a standard neurological examination was administered to each patient, not all signs and symptoms may have been documented from the clinical interview with the patient and informant. This may lead to an underestimation of the true prevalence of apraxia and associated features. Due to the retrospective nature of this study, standardized tests of praxis were not included in the assessment to determine the sensitivity of routine clinical examination, and further research is warranted to examine this. Finally, the sample sizes in some of the dementia subtypes were small. Although this reflects the relative rarity of some syndromes, replication of these findings is needed in a larger sample.

Despite these limitations, the strength of this study is in highlighting that simple and routine examination can provide pointers to diagnosis at initial clinical presentation, with an average of 2 years symptom duration. Our results suggest that there exist disease-specific profiles of limb apraxia and associated deficits, and that these are clinically relevant in the differential diagnosis of AD and FTD spectrum disorders. Our findings have the potential to inform future diagnostic protocols and warrant further investigation into more detailed, quantitative assessment of these features.
